# Serotonergic Psychedelics Temporarily Modify Information Transfer in Humans

**DOI:** 10.1093/ijnp/pyv039

**Published:** 2015-04-27

**Authors:** Joan Francesc Alonso, Sergio Romero, Miquel Àngel Mañanas, Jordi Riba

**Affiliations:** Biomedical Engineering Research Centre, Department of Automatic Control (Drs Alonso, Romero, and Mañanas), and; Barcelona College of Industrial Engineering (Drs Alonso and Mañanas), Universitat Politècnica de Catalunya, Barcelona, Spain;; CIBER de Bioingeniería, Biomateriales y Nanomedicina, Spain (Drs Romero and Mañanas);; Human Neuropsychopharmacology Group, Sant Pau Institute of Biomedical Research, Barcelona, Spain (Dr Riba);; Department of Pharmacology and Therapeutics, Universitat Autònoma de Barcelona, Barcelona, Spain (Dr Riba);; Centro de Investigación Biomédica en Red de Salud Mental, CIBERSAM, Spain (Dr Riba).

**Keywords:** Oscillatory brain dynamics, psychedelics, functional connectivity, transfer entropy, human

## Abstract

**Background:**

Psychedelics induce intense modifications in the sensorium, the sense of “self,” and the experience of reality. Despite advances in our understanding of the molecular and cellular level mechanisms of these drugs, knowledge of their actions on global brain dynamics is still incomplete. Recent imaging studies have found changes in functional coupling between frontal and parietal brain structures, suggesting a modification in information flow between brain regions during acute effects.

**Methods:**

Here we assessed the psychedelic-induced changes in directionality of information flow during the acute effects of a psychedelic in humans. We measured modifications in connectivity of brain oscillations using transfer entropy, a nonlinear measure of directed functional connectivity based on information theory. Ten healthy male volunteers with prior experience with psychedelics participated in 2 experimental sessions. They received a placebo or a dose of ayahuasca, a psychedelic preparation containing the serotonergic 5-HT_2A_ agonist *N,N*-dimethyltryptamine.

**Results:**

The analysis showed significant changes in the coupling of brain oscillations between anterior and posterior recording sites. Transfer entropy analysis showed that frontal sources decreased their influence over central, parietal, and occipital sites. Conversely, sources in posterior locations increased their influence over signals measured at anterior locations. Exploratory correlations found that anterior-to-posterior transfer entropy decreases were correlated with the intensity of subjective effects, while the imbalance between anterior-to-posterior and posterior-to-anterior transfer entropy correlated with the degree of incapacitation experienced.

**Conclusions:**

These results suggest that psychedelics induce a temporary disruption of neural hierarchies by reducing top-down control and increasing bottom-up information transfer in the human brain.

## Introduction

Growing interest in psychedelics or “mind-manifesting” drugs as models of disease (Kometer et al., 2011; Carhart-Harris et al., 2013) and potential therapeutic agents in psychiatry (Sessa, 2005; Grob et al., 2011) has stimulated new research into their neural mechanisms. Evidence of their modulatory capacity on the amygdala and ACC suggests their potential efficacy in the treatment of affective disorders (Vollenweider and Kometer, 2010; Kraehenmann et al., 2014). One of the psychedelics whose use has expanded most rapidly in recent years is ayahuasca. This plant-derived preparation contains *N,N*-dimethyltryptamine (DMT), a classical psychedelic with 5-HT_2A_ agonist properties (González-Maeso and Sealfon, 2009). DMT is structurally related to the neurotransmitter serotonin (5-hydroxytryptamine) and induces brief but intense modifications of the ordinary state of awareness (Strassman et al., 1994). Ayahuasca, which contains natural monoamine-oxidase inhibitors, induces effects that are more prolonged in time, reaching their maximum intensity around 1.5 and 2 hours after administration (Riba et al., 2001b, 2003; Dos Santos et al., 2011).

Recent studies using neurophysiological and neuroimaging techniques have advanced our knowledge of the brain areas targeted by psychedelics. Two major brain hubs have been implicated in the effects of these drugs: the medial aspects of the frontal cortex including the anterior cingulate cortex (ACC) and several regions in the parietal lobes, including the posterior cingulate cortex and dorsolateral parietal cortex (Riba et al., 2004, 2006; Carhart-Harris et al., 2012; Muthukumaraswamy et al., 2013; Palhano-Fontes et al., 2015). Nuclear medicine techniques such as PET and SPECT have found increases in glucose metabolism and blood flow essentially at the frontal level after administration of the serotonergic psychedelics mescaline (Hermle et al., 1992), psilocybin (Vollenweider et al., 1997), and ayahuasca (Riba et al., 2006). These studies, however, showed few or no effects at more posterior regions.

Drug-induced changes in parieto-occipital areas have been evidenced using electrophysiological measures. Studies of ayahuasca-induced changes in brain oscillations (EEG) have found decreases in current density in frontomedial regions. However, more intense and widespread reductions have been located in the parietal cortex in an area centered on the posterior cingulate (Riba et al., 2004). Using magnetoencephalography (MEG), analogous power reductions of magnetic signals in the posterior cingulate and adjoining parietal areas have been found following intravenous psilocybin (Muthukumaraswamy et al., 2013).

Interactions between frontal and posterior areas have been described only recently. An MRI study of resting state has shown that acute administration of psilocybin decreases functional coupling between prefrontal and parietal cortical midline regions, including the anterior and posterior cingulate cortices (Carhart-Harris et al., 2012). Furthermore, long-term use of ayahuasca has been associated with opposite changes in brain structure in the anterior and posterior cingulate cortices in humans (Bouso et al., 2015).

The aim of this study was to assess the psychedelic-induced changes in the dynamic interaction of brain oscillations and the directionality of drug-induced modifications. We postulated that psychedelics modify neural hierarchies and the flow of information around the brain. To test this hypothesis, we studied directed functional connectivity of brain oscillations using transfer entropy (TE). This nonlinear measure identifies causal relationships, enabling the inference of directionality in the observed changes.

## Materials and Methods

### Participants and Drug Administration

We recruited a group of 10 healthy male volunteers with previous experience in psychedelic drug use. Prior to participation, all volunteers underwent a medical examination that included anamnesis, ECG, and standard laboratory tests to confirm good health. After being informed about the nature of ayahuasca and its general psychological and potential adverse effects, all volunteers gave their written informed consent to participate. The study was conducted in accordance with the Declaration of Helsinki and subsequent amendments concerning research in humans and was approved by the Hospital Ethics Committee and the Spanish Ministry of Health.

The study was conducted according to a double-blind, randomized, balanced crossover design. EEG recordings, subjective effects measures, and plasma samples were obtained in 2 experimental sessions 1 week apart. In one of the sessions, participants received a placebo and in another session they received an oral dose of ayahuasca. Ayahuasca was not administered in liquid form but as a freeze-dried formulation. Following lyophilization, ayahuasca was homogenized, analyzed for alkaloid contents, and encapsulated. The dose administered was equivalent to 0.75mg DMT/kg body weight and was chosen as a medium-intensity dose based on a previous study (Riba et al., 2001b). Treatments were administered in a double-blind randomized fashion.

### EEG Recording and Processing

Three-minute EEG recordings with eyes closed were recorded from 19 standard scalp leads (Fp1/2, F3/4, Fz, F7/8, C3/4, Cz, T3/4, T5/6, P3/4, Pz and O1/2). Recordings were obtained using a NeuroScan SynAmps amplifier (Compumedics Neuroscan, Charlotte, NC) before drug intake (baseline) and at 15, 30, and 45 minutes, and 1, 1.5, 2, 2.5, 3, and 4 hours after administration. Signals were referenced to the averaged mastoid electrodes, and vertical and horizontal electrooculograms were also obtained for artifact minimization and rejection. Signals were analogically band-pass filtered between 0.1 and 45 Hz, digitized with a frequency of 250 Hz, and subsequently digitally resampled to 100 Hz.

EEG artifact minimization and rejection was performed prior to parameter calculation. This was carried out following a previously described method based on blind source separation (Romero et al., 2008; Alonso et al., 2010). Subsequently, a 60-second segment was cropped out and zero-phase filtered between 0.5 and 35 Hz using a type-II Chebyshev filter of order 8.

### TE Calculation

Following artifact reduction, TE was calculated. TE from *y* to *x* measures the amount of uncertainty reduced in the future values of *x* by taking into account the past values of *y* compared with when only the past values of *x* are used. Considering a delay of one sample in a time series (Schreiber, 2000), TE measures the increase in information obtained at *x*_*n+1*_ when *x*_*n*_ and *y*_*n*_ are known compared with the situation when only *x*_*n*_ is known.

The information carried by a variable can be calculated by means of the Shannon entropy. If we consider 2 random variables, *X* and *Y*, with probability distribution functions *p(x*) and *p(y*) and the joint probability distribution function *p(x,y*), the Shannon entropy of *X* can be expressed as: 

$$H(X)=-\sum_{x}p(x)log\left(p(x)\right)$$ (1)

The Shannon entropy can be measured in bits if the base of the logarithm is 2. The joint Shannon entropy of the 2 variables will be: 

$$H(X,Y)=-\sum_{x,y}p(x,y)log\left(p(x,y)\right)$$ (2)

To obtain the expression of TE, we can consider the amount of additional information required to represent the value of a future observation of a signal by defining the following entropy rate for 2 signals: 

$$\begin{array}{l} h=-\sum_{x_{n+1}}p\left(x_{n+1},x_{n},y_{n}\right)log\left(p\left(x_{n+1}|x_{n},y_{n}\right)\right)=\\ \;-\sum_{x_{n+1}}p\left(x_{n+1},x_{n},y_{n}\right)log\left(\frac{p\left(x_{n+1},x_{n},y_{n}\right)}{p\left(x_{n},y_{n}\right)}\right) \end{array}$$ (3)

If we consider *x*_*n+1*_ independent from *y*_*n*_, equation 3 can be written as: 

$$\begin{array}{l} h=-\sum_{x_{n+1}}p\left(x_{n+1},x_{n},y_{n}\right)log\left(p\left(x_{n+1}|x_{n}\right)\right)=\\ \;-\sum_{x_{n+1}}p\left(x_{n+1},x_{n},y_{n}\right)log\left(\frac{p\left(x_{n+1},x_{n}\right)}{p\left(x_{n}\right)}\right) \end{array}$$ (4)

TE is obtained by subtracting equation 4 from equation 3: 

$$\begin{array}{c} TE_{y\to x}=\sum_{x_{n+1}}p\left(x_{n+1},x_{n},y_{n}\right)log\left(p\left(x_{n+1}|x_{n},y_{n}\right)\right)\\ -\sum_{x_{n+1}}p(x_{n+1},x_{n},y_{n})log\left(p\left(x_{n+1}|x_{n}\right)\right)\\ =\sum_{x_{n+1}}p\left(x_{n+1},x_{n},y_{n}\right)log\left(\frac{p\left(x_{n+1}|x_{n},y_{n}\right)}{p\left(x_{n+1}|x_{n}\right)}\right)\\ =\sum_{x_{n+1}}p\left(x_{n+1},x_{n},y_{n}\right)log\left(\frac{p\left(x_{n+1},x_{n},y_{n}\right)p\left(x_{n}\right)}{p\left(x_{n},y_{n}\right)p\left(x_{n+1},x_{n}\right)}\right) \end{array}$$ (5)

TE is inherently asymmetric, so that we can analogously define $TE_{x\to y}$: 

$$TE_{x\to y}=\sum_{x_{n+1}}p\left(y_{n+1},y_{n},x_{n}\right)log\left(\frac{p\left(y_{n+1},x_{n},y_{n}\right)p\left(y_{n}\right)}{p\left(x_{n},y_{n}\right)p\left(y_{n+1},y_{n}\right)}\right)$$ (6)

TE was estimated using a nonparametric methodology based on equiquantal binning, that is, estimating the probability distribution functions using marginal equiquantization and histograms. This procedure is recommended when sufficient data points are available (Vejmelka and Palus, 2008; Lee et al., 2012). While Lee et al. (2012) showed better results using KDE or D-V partitioning when fewer than 200 data points are used, our 60-second signals provided 6000 data points and probability distributions could be estimated reliably.

### Subjective Effect Measures and Blood Samples

The time course of subjective effects was measured using a 100-mm visual analog scale. Participants were asked to rate the overall intensity of effects by drawing a vertical line crossing the visual analog scale. This instrument was administered immediately before the administration of ayahuasca or placebo (baseline) and at 15, 30, and 45 minutes, and 1, 1.5, 2, 2.5, 3, and 4 hours after dosing.

Additionally, volunteers were also requested to answer 2 questionnaires measuring psychedelic-induced subjective effects. The first questionnaire was a Spanish version of the Hallucinogen Rating Scale (HRS) (Riba et al., 2001a). The HRS includes 6 subscales: somaesthesia, reflecting somatic effects; affect, sensitive to emotional and affective responses; volition, indicating the incapacitation experienced; cognition, describing modifications in thought processes or content; perception, measuring visual, auditory, gustatory, and olfactory experiences; and intensity, which reflects the strength of the overall experience.

The second questionnaire administered was a Spanish version of the Altered States of Consciousness Questionnaire (Aussergewöhnliche Psychische Zustände [APZ]) developed by Dittrich (1998). It includes 3 subscales: oceanic boundlessness (Ozeanische Selbstentgrenzung [OSE]), measuring changes in the sense of time, derealization, and depersonalization phenomena; dread of ego-dissolution (Angstvolle IchAuflösung [AIA]), measuring thought disorder and decreased body and thought control associated with anxiety; and visionary restructuralization (Visionäre Umstrukturierung [VUS]), measuring illusions, hallucinations, and synesthesia.

Participants completed the HRS and APZ at 4 hours following drug administration once the psychoactive effects of ayahuasca had worn off.

Blood samples were obtained immediately prior to drug administration (baseline) and at 0.5, 1, 1.5, 2, 2.5, 3, and 4 hours thereafter. DMT plasma concentrations were determined as previously published (Yritia et al., 2002).

### Statistical Analysis

To investigate potential changes in TE induced by ayahuasca, a total of 19*18=342 connections were introduced in the analysis. To obtain the net pharmacological effect, the TE values obtained before drug intake (placebo or ayahuasca) were subtracted from post-drug values. Statistical differences between placebo and ayahuasca were then calculated.

Two-sided Wilcoxon signed-rank tests were carried out to search for differences at the different time-points. The results from the statistical tests were graphically presented using schematic representations of electrodes on the scalp. Statistically significant comparisons between EEG leads were shown by connections between the corresponding electrodes. In these connection maps, warm colors indicated significant increases and cold colors indicated significant decreases. Thick, dark-colored connections indicated statistically significant changes with *P*<.01 uncorrected, whereas thick and thin light-colored connections indicated *P*<.05 and .1, respectively. Connection maps were analyzed separately for increases and decreases. To assess the statistical significance of changes in directed connectivity, we used these uncorrected thresholds, and then the significance of the functional disintegration over all connections was assessed using an omnibus statistic (the number of above threshold changes in directed connectivity). This omnibus test has been widely used in many studies in several fields such as primatology (Muniz et al., 2010), ecology (Verfuß et al. 2006), neurology, and pharmacology (Saletu et al., 2004, 2005; Alonso et al., 2010, Painold et al., 2011), among others. This statistic followed a binomial distribution under the null hypothesis of no changes anywhere (Cross and Chaffin, 1982). Thus, uncorrected *P*-values were treated as normalized effect sizes, but the multiple comparison problem was solved by reporting changes only when their number was significant. The connection maps shown in the results section were only those with a number of significant changes (connections) exceeding the calculated threshold that was set at 44 according to the binomial criterion.

As TE is a directional measure, directionality maps were drawn showing the number of incoming or outgoing connections for each electrode. Thus, for each schematic representation showing increases and decreases of TE, 2 additional directionality maps were drawn to show sources (outgoing information, green) and sinks (incoming information, gray). In each case, a color scale indicated intensity. The darkest tone (either green or gray) indicated 7 or more connections going in or out of the corresponding electrode and white indicated no connections.

These directionality maps, which indicated the origin and destination areas of the transferred information, were only calculated if the prior connection maps were considered significant according to the uncorrected *P*-value.

Scores on the HRS and APZ questionnaires were compared between placebo and ayahuasca using Student’s *t* tests followed by correction for multiple comparisons using the false discovery rate. Results were considered significant for false discovery rate corrected *P*<.05.

Finally, data were explored for correlations between TE changes and subjective data (ayahuasca scores - placebo scores) and DMT plasma levels. Correlations were calculated using Pearson’s correlation coefficient. Given the exploratory nature of this analysis, no correction for multiple comparisons was applied here.

## Results

### EEG Signals, TE

**Figure 1.**
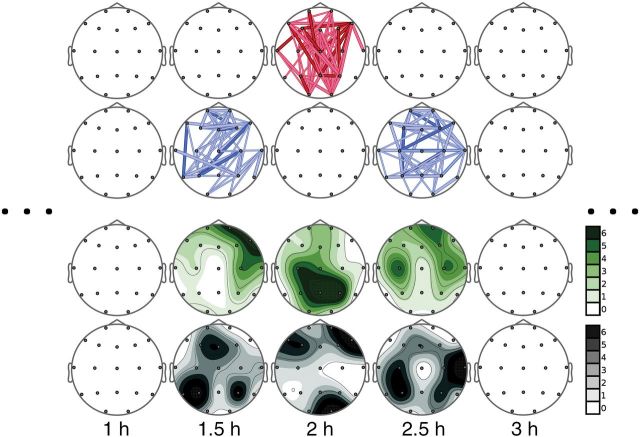
Results of the statistical test are shown on schematic representations of electrodes on the scalp. Significant changes are shown by connections between the corresponding electrodes. Warm colors indicate significant increases (first row) and cold colors (second row) indicate significant decreases. Thick, dark-colored connections indicate statistically significant changes with *P*<.01, whereas thick and thin light-colored connections indicate *P*<.05 and 0.1, respectively. Connections are shown only if their number in the connections map was above the threshold set by the binomial criterion. Otherwise, no connections are depicted, because they are considered a random result not due to drug administration (see text). The connection maps for the time points that are not shown had a lower number of significant connection changes, and they were considered not relevant based on the binomial criterion. The third and fourth rows show the directionality maps depicting the number of outgoing (in green) or incoming (in gray) connections for each electrode, respectively. In each case, a 7-level color scale indicates intensity. Note that the darkest tone indicates 6 or more connections going in or out of the corresponding electrode, and white indicates no connections.

Compared with placebo, ayahuasca induced significant TE changes at 1.5, 2, and 2.5 hours following drug administration (Figure 1). As shown in the connection maps, significant increases of TE were observed for many electrode pairs at 2 hours after ayahuasca administration. These increases were preceded and followed at 1.5 and 2.5 hours, respectively, by significant decreases.

The directionality of change is shown in the figure below the connection maps. Topographic representations of the source and sink areas show that TE increases at 2 hours were governed by information originating at posterior leads (in green). This information mainly influenced signals recorded at more frontal locations (in gray). In other words, uncertainty at frontal locations decreased, that is, predictability increased when information at posterior locations was taken into account. In contrast, the topographic maps corresponding to 1.5 and 2.5 hours showed that the TE decreases were caused by a reduced influence of information at anterior leads (in green) over signals at more posterior locations (in gray). In terms of predictability, posterior activity became significantly less predictable when anterior signals were taken into account. The pattern of anterior-to-posterior TE decreases and posterior-to-anterior TE increases was observed at all time points between 45 minutes and 4 hours following drug administration. However, statistical significance was reached only at the time points mentioned above. In the supplementary Material file, we include the directionality maps for all time-points irrespective of statistical significance. This approach showed that the pattern of ayahuasca-induced changes in TE was consistent throughout the duration of ayahuasca effects.

### Subjective Effects and DMT Plasma Levels

**Figure 2.**
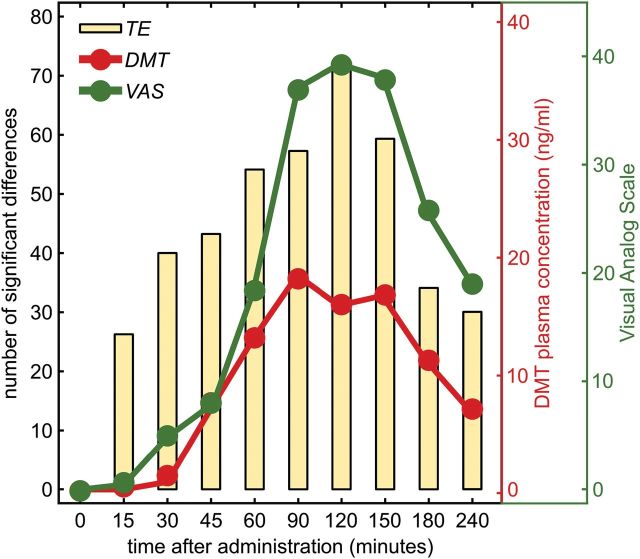
Time course of the number of significant changes induced by ayahuasca administration detected by transfer entropy (TE; yellow bars). The red and green traces show the *N,N*-dimethyltryptamine (DMT) concentration and visual analog scale scores, respectively.

As shown in Figure 2, parallel variations were observed between TE, the time course of subjective effects, and DMT plasma concentrations. The maximum values for each of these measures were observed between 1.5 and 2.5 hours following administration. Scores on the HRS and APZ questionnaires are shown in Table 1. Compared with placebo, ayahuasca induced statistically significant increases in all 6 subscales of the HRS. In the APZ, the VUS subscales were significantly increased, while nonsignificant elevations were observed in the OSE and AIA subscales.

**Table 1.** Subjective Effects Induced by Placebo and Ayahuasca (0.75mg DMT/kg Body Weight)

**HRS**	**Placebo**	**Ayahuasca**	**Student’s *t* Test**		**Significance** **(FDR Corrected)**
			**t value**	***P* value**	
Somaesthesia	0.00 (0.00)	1.15 (1.01)	-3.56	.006	*
Affect	0.27 (0.06)	1.27 (0.90)	-3.52	.007	*
Perception	0.00 (0.00)	1.28 (1.14)	-3.56	.006	*
Cognition	0.00 (0.00)	1.21 (1.09)	-3.52	.007	*
Volition	0.33 (0.50)	1.11 (0.56)	-3.92	.003	*
Intensity	0.10 (0.32)	1.93 (1.03)	-5.98	.000	*
APZ					
OSE	0.10 (0.32)	4.00 (4.27)	-2.88	.018	n.s.
AIA	0.00 (0.00)	3.40 (5.60)	-1.92	n.s	n.s.
VUS	0.00 (0.00)	5.60 (5.21)	-3.40	.008	*

Abbreviations: AIA, dread of ego dissolution; APZ, Aussergewöhnliche Psychische Zustände; FDR, false discovery rate; HRS, Hallucinogen Rating Scale; OSE, oceanic boundlessness; VUS, visionary restructuralization.

Means (SD) of the scores obtained for the HRS and APZ questionnaires and results of the statistical analysis. n=10. Results were corrected for multiple comparisons using the FDR, which yielded a threshold value for significance of *P=*.0180.

*Significant following FDR correction, n.s., not significant.

### Correlation Analysis

**Figure 3.**
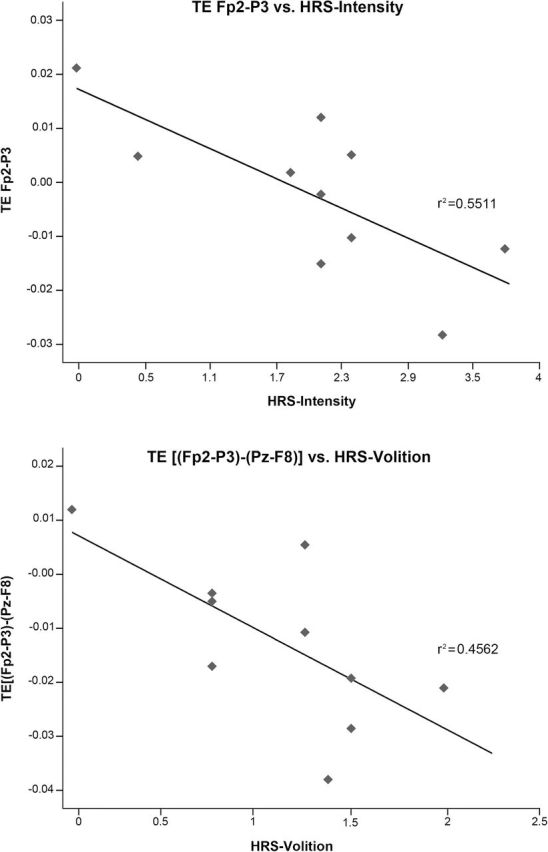
Scatter plots showing the relationship between mean transfer entropy (TE) values for the time period 1.5 to 2.5 hours and Hallucinogen Rating Scale (HRS) scores.

Mean TE values for the time period 1.5 to 2.5 hours were calculated for electrode pairs that showed statistically significant differences from placebo. These mean values were correlated with scores on the HRS and APZ questionnaires. Significant correlations were found for TE decreases, for example, in the FP2→P3 pair and the HRS scales perception (r=-0.640, *P*=.046), cognition (r=-0.665, *P*=.036), and intensity (r=-0.736, *P*=.015). Also between TE decreases in the FP2→P3 pair and the APZ scales OSE (r=-0.705, *P*=.023), AIA (r=-0.684, *P*=.029), and VUS (r=-0.678, *P*=.031) (see Figure 3). No correlations were found for posterior to anterior TE increases. We subsequently tested whether the ayahuasca-induced imbalance between anterior-to-posterior and posterior-to-anterior information transfer was associated with the subjective experience. To do so, we calculated the difference values between anterior-to-posterior and posterior-to-anterior TE for the different electrode pairs (for instance, the difference value (Fp2→P3)-(Pz→F8)). We found that values calculated this way were significantly correlated with the HRS volition scale: [(Fp2→P3)-(Pz→F8); r=-0.666, *P*=.036], and [(Fp2→P3)-(Pz→F7); r=-0.654, *P*=.040].

### Data Reanalysis Using Granger Causality

To assess the robustness of the TE findings, we conducted a reanalysis of the data at the 2-hour time point using Granger Causality (GC). TE is a model-free generalization of GC and is equivalent to the latter for data following a normal distribution (Barnett et al., 2009). However, GC is a more commonly used approach to functional connectivity in the field of neuroscience. Its mathematical formulation is based on linear regression modeling of stochastic processes (Granger, 1969).

GC calculations were implemented using the MVGC multivariate GC toolbox (Barnett and Seth, 2014) using the work-flow recommended by the authors. An optimal model order of 8 was used (according to the Akaike Information Criterion), although we also tested orders 4 and 16 to look for possible differences regarding the VAR model estimation. The resulting causalities were evaluated following the experimental design (baseline correction and compared with placebo).

**Figure 4.**
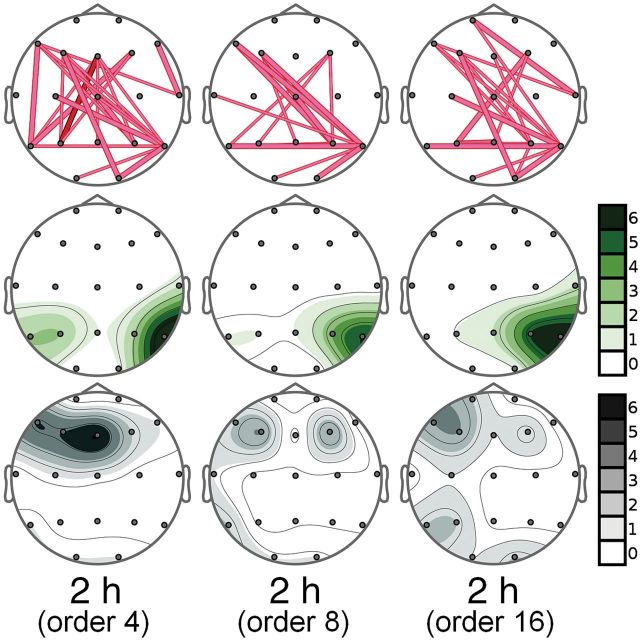
Reanalysis of functional connectivity at the 2-hour time point using Granger Causality (GC) with model orders of 4, 8, and 16. Significant changes are shown in the first row by lines connecting the corresponding electrodes and indicate increases. Thick, dark lines indicate statistically significant increases with *P*<.01, whereas thick and thin light-colored lines indicate increases with *P*<.05 and 0.1, respectively. The second and third rows show the directionality maps showing the number of outgoing (green) or incoming (gray) connections for each electrode, respectively. In each case, a 7-level color scale indicates intensity. Note that the darkest tone indicates 6 or more connections going in or out of the corresponding electrode, and white indicates no activity.

The results of the reanalysis are shown in Figure 4. Significant increases in postero-anterior connections were observed, although their number was lower than with TE. The number of significant connections also varied slightly between model order, but the overall pattern of results is analogous to our original analysis, supporting the conclusions reached using TE.

## Discussion

The directed functional connectivity analysis reported here showed psychedelic-induced changes in antero-posterior coupling of electrophysiological brain oscillations. Acute administration of ayahuasca altered brain dynamics, as measured by TE. As opposed to other connectivity measures such as coherence or mutual information, TE identifies directed causal relationships enabling the inference of directionality in the observed changes. Note that a pair-wise estimate of TE was used, meaning that information flow from all other regions was not taken into account when considering the directed functional connectivity between any 2 nodes. In other words, it is possible that some of the reproduced dependencies may have varied if information transfer from other nodes had been taken into account. However, this does not really matter, because our primary results stand in terms of directed statistical dependencies and their changes due to psychopharmacological effects.

Results showed that ayahuasca acutely decreased anterior-to-posterior information transfer and increased posterior-to-anterior information transfer. These neurophysiological changes ran parallel to DMT plasma concentrations and subjective effects. These findings suggest that psychedelics transiently disrupt neural hierarchies by modifying information transfer between brain regions. These results were further supported by a reanalysis of the data using GC. Again, posterior-to-anterior connectivity increases were observed at the 2-hour time point. However, the number of significant connections was lower than with TE, possibly because of the length of the signals and minor local nonstationarities, to which the equiquantal binning-based estimation of TE is quite robust.

The present results combine the high temporal resolution of neurophysiological measures with the directionality information provided by TE and add to anatomical data available from neuroimaging techniques. Various studies have highlighted the medial aspects of the frontal and parietal cortices as key structures involved in the modified states of awareness induced by psychedelics. Nuclear medicine studies using PET and SPECT have emphasized increased blood perfusion and glucose metabolism in several frontomedial regions, including the ACC. Such increases have been observed following the classic serotonergic psychedelics mescaline and psilocybin (Hermle et al., 1992; Vollenweider et al., 1997) and after subanesthetic doses of the glutamatergic NMDA receptor antagonist ketamine (Daumann et al., 2010). Similarly, in a previous SPECT study by our group, we also found increases in blood flow in frontomedial regions after acute ayahuasca administration (Riba et al., 2006).

Despite converging evidence from the various radiotracer studies, analysis of drug-induced changes in brain oscillations has yielded only partially overlapping results. Source localization studies of ayahuasca-induced changes on the EEG found significant modifications in current density in the ACC and neighboring areas, but also in parietal and occipital cortex (Riba et al., 2004). Here, we have replicated these results in a current density analysis that we report in the supplementary Figure S2 and associated text. Thus, we found broadband reductions in current density in medial posterior (precuneus and cuneus) and anterior (cingulate) brain areas. A recent MEG study has extended these findings to psilocybin and magnetic signals. The results highlighted the parietal cortex as the main site of broadband oscillatory decreases acutely induced by the drug (Muthukumaraswamy et al., 2013). These seemingly paradoxical effects involving energy decreases in EEG and MEG and simultaneously enhanced visual activity can be interpreted in light of multiple-technique studies. An early study involving topographic electroencephalography and PET already found an inverse relationship between alpha power and cerebral glucose use (Buchsbaum et al., 1984). More recently, combined studies using BOLD and EEG found a negative correlation between alpha and BOLD in the anterior cingulate and parieto-occipital cortex, especially in visual areas (Goldman et al., 2002; Laufs et al., 2003; de Munck et al., 2007). Thus, a decreased alpha rhythm has been associated with metabolic activation (Moosmann et al., 2003). These findings have recently been extended to other frequency bands of the EEG, including theta and beta (de Munck et al., 2009). The observed broad-band power decreases found after ayahuasca in the current source density analysis can be interpreted as reflecting increased activation of areas involved in visual processing (occipital cortex) and cognitive-emotional processing (ACC).

TE results in the present study show that functional coupling of EEG signals is altered in the psychedelic state and predominantly so along the anterior-to-posterior axis. Our analysis of directed functional connectivity using TE shows the involvement of both the anterior and posterior brain, combining the partial findings of the more classical techniques mentioned above. Our analysis shows that psychedelics temporarily modify the dynamics of the interaction between the higher order frontal regions and the more sensory-selective posterior areas. In line with our TE data, a study in rodents has also shown psychedelic-induced changes in the functional coupling of EEG signals between frontal and parietal sites. The psychedelic phenethylamine 2C-B increased EEG connectivity in these animals (Páleníček et al., 2013). In another study, increased coherence was reported in the high-frequency bands after ayahuasca administration (Stuckey et al., 2005). Additional evidence of modified functional coupling between frontal and parietal regions in the psychedelic state was recently provided by a magnetic resonance imaging study of psilocybin effects in humans (Carhart-Harris et al., 2012). Following the intravenous administration of the drug, the authors reported increases in BOLD signal coupling between the anterior and posterior cingulate cortices. Their approach, however, did not provide information on potential changes in the direction of information flow.

Inference of directionality can be made with TE. Our data indicate a decrease in the predictability of activity in posterior areas based on information available at anterior sites and, conversely, an increase in the predictability of activity in anterior areas when information at posterior sites is taken into account. In other words, the hierarchies governing the flow of information observed after placebo are transiently disrupted by psychedelics. These findings can be interpreted in light of emerging theoretical views of brain function. Bayesian inference models postulate that the subjective experience of reality emerges from the dynamic interaction between bottom-up or feed-forward sensory information and top-down or feedback projections that interpret incoming signals based on previous knowledge and expectations (Friston, 2005; Mesulam, 2008). In this theoretical framework, the frontal cortex is at the top of the hierarchy, sending backward projections that modulate incoming information according to a series of constraints. This particular finding of asymmetrical changes in top-down connections from the prefrontal cortex is interesting in relation to the findings of Boly et al. (2011), who used dynamic causal modelling to show a selective impairment of top-down frontal connectivity in impaired consciousness.

We speculate that when top-down constraints are reduced and sensory excitability is increased (de Araujo et al., 2012), weak endogenous activity or “system noise” in visual and auditory cortices is then able to reach higher levels in the hierarchy. This could explain the visual phenomena commonly reported for psychedelics. These visual effects are typically more prominent with eyes closed when “system noise” does not have to compete with strong external visual stimuli (Riba et al., 2001b). Higher excitability at the posterior heteromodal association cortex, the cingulate, and other paralimbic structures (Riba et al., 2004, 2006) could explain the “seepage” of information between sensory modalities causing synesthesia and the high emotional lability induced by psychedelics (Riba et al., 2001b). In the study by Muthukumuraswarmy and coworkers (2013), dynamic causal modelling revealed that posterior cingulate desynchronization can be explained by increased excitability of deep layer pyramidal neurons, which are known to be rich in 5HT_2A_ receptors. This fits comfortably with our findings in relation to TE in the sense that the posterior regions come to dominate information outflow to other (eg, prefrontal) regions. This higher excitability combined with the loosening of the cognitive grip exerted by frontal regions responsible for executive control may underlie the overwhelming nature of the experience. In addition to drug potency and dose, we can postulate that these interactions will also be modulated by factors affecting each individual, such as mood, personality, expectations, and prior experience with psychedelic drugs. Thus, the perceptual modifications, associations, and insights that emerge will not only vary between individuals but also from one intake to the next, making each psychedelic experience novel and surprising (Shanon, 2002).

It can be argued that if this disruption of hierarchies were to linger after discontinuation of the drug, problems could emerge in the normal processing of sensory information. Longer-lasting changes at this level could explain some of the adverse consequences associated with psychedelic drug use. The hallucinogen persistent perception disorder, an infrequent but highly distressing disorder (Halpern and Pope, 2003), could reflect a chronic impairment of functional coupling. In a study examining EEG connectivity in chronic LSD users suffering from hallucinogen persistent perception disorder, the authors found increased intra-occipital coherence and decreased coherence between posterior and frontal sites (Abraham and Duffy, 2001). Additionally, they reported a decreased latency of the P200 component of the visual evoked potential. The authors had previously speculated that pathological disinhibition of posterior cortical activity could be responsible for the visual phenomena reported by the patients (Abraham and Duffy, 1996).

To sum up, acute administration of a psychedelic disrupted the functional connectivity of brain oscillations in humans. TE measures showed that signals at frontal locations decreased their influence over oscillations at more posterior sites. Conversely, sources in posterior locations increased their influence over signals measured at anterior locations. Drug-induced modifications in TE were associated with the intensity and time course of subjective effects. These results suggest that psychedelics induce a temporary disruption of neural hierarchies governing the flow of information by reducing top-down control and enhancing bottom-up information transfer. These changes could underlie the profound modifications of perception, cognitive processes, and experience of reality typically induced by these drugs.

## Interest Statement

None.

## Supplementary Material

supplementary Figure S2
