# Erratum

**DOI:** 10.1093/ijnp/pyv099

**Published:** 2015-09-08

Alonso JF, Romero, S, Mañanas MÀ, Riba J (2015) Genotypes Do Not Confer Risk For Serotonergic Psychedelics Temporarily Modify Information Transfer in Humans. Int J Neuropsychopharmacol 18(8). doi: 10.1093/ijnp/pyv039.

In Volume 18, Issue 8 of *The International Journal of Neuropsychopharmacology*, DOI: 10.1093/ijnp/pyv039, the following corrections were not made:

1. Drs Alonso, Romero, and Mañanas are all affiliated with CIBER de Bioingeniería, Biomateriales y Nanomedicina, Spain.
2. On page 3 in the Statistical Analysis section, the following sentence did not include the correct in-text citation, Verfuß et al. 2007, but instead listed the year as 2006: “This omnibus test has been widely used in many studies in several fields such as primatology (Muniz et al., 2010), ecology (Verfuß et al. 2006), neurology, and pharmacology (Saletu et al., 2004, 2005; Alonso et al., 2010, Painold et al., 2011), among others. This statistic followed a binomial distribution under the null hypothesis of no changes anywhere (Cross and Chaffin, 1982).”
3. The following references were not included:

Muniz L, Perry S, Manson JH, Gilkenson H, Gros-Louis J, Vigilant L (2010) Male dominance and reproductive success in wild white-faced capuchins (*Cebus capucinus*) at Lomas Barbudal, Costa Rica. Am J Primatol 72(12). doi: 10.1002/ajp.20876.

Painold A, Anderer P, Holl AK, Letmaier M, Saletu-Zyhlarz GM, Saletu B, Bonelli RM (2011) EEG low-resolution brain electromagnetic tomography (LORETA) in Huntington**’**s disease. J Neurol 258(5). doi: 10.1007/s00415-010-5852-5.

Saletu M, Anderer P, Saletu-Zyhlarz GM, Mandl M, Arnold O, Zeitlhofer J, Saletu B (2004) EEG-tomographic studies with LORETA on vigilance differences between narcolepsy patients and controls and subsequent double-blind, placebo-controlled studies with modafinil. J Neurol 251(11). doi: 10.1007/s00415-004-0543-8.

Saletu MT, Anderer P, Saletu-Zyhlarz GM, Mandl M, Arnold O, Nosiska D, Zeitlhofer J, Saletu B (2005) EEG–mapping differences between narcolepsy patients and controls and subsequent double–blind, placebo–controlled studies with modafinil. Eur Arch Psychiatry Clin Neurosci 255(1). doi: 10.1007/s00406-004-0530-1.

Verfuß UK, Honnef, CG, Meding A, D ä hne M, Mundry R, Benke H (2007) Geographical and seasonal variation of harbour porpoise (*Phocoena phocoena*) presence in the German Baltic Sea revealed by passive acoustic monitoring. J Mar Biol Assoc U.K. 87(1). doi: 10.1017/S0025315407054938.

4. The study was support by the Ministerio de Ciencia e Innovación under contracts DPI 2011–22680 and DPI2014-59049-R.

The publisher regrets this error.

