# Supplementary material for: Serotonergic Psychedelics Temporarily Modify Information Transfer in Humans
Source: Int J Neuropsychopharmacol. 2015 Apr 27;18(8):pyv039. doi: 10.1093/ijnp/pyv039 (PMC4571623; doi:10.1093/ijnp/pyv039)
Supplement: supplementary Figure S2 [file Supplementary_material_rev_FINAL.doc]

**Supplementary material**

**Figure S1:** Figure S1 shows that the effects of ayahuasca on TE were present at most time points. These effects involved: a) an increase in information transfer from posterior to anterior brain regions, and b) a decrease in information transfer from anterior to posterior brain regions. The more stringent statistical threshold used in Figure 1 (main text) can lead to the false impression that opposite effects take place at the different time points. The maps in Figure 1 show that posterior-to-anterior increases in TE predominate at 2h and anterior-to-posterior decreases in TE predominate at 1.5 and 2.5 h. However, posterior-to-anterior increases in TE and anterior-to-posterior decreases in TE occurred from 45 minutes onwards. This can be visualized below in directionality maps showing all sources and sinks irrespective of statistical significance.

a) Directionality maps showing **increases** in TE at all measured time points. Sources of increase are depicted in green and sinks in gray-black. Note the predominance of posterior locations for sources between 45 min and 4 hours after dosing.


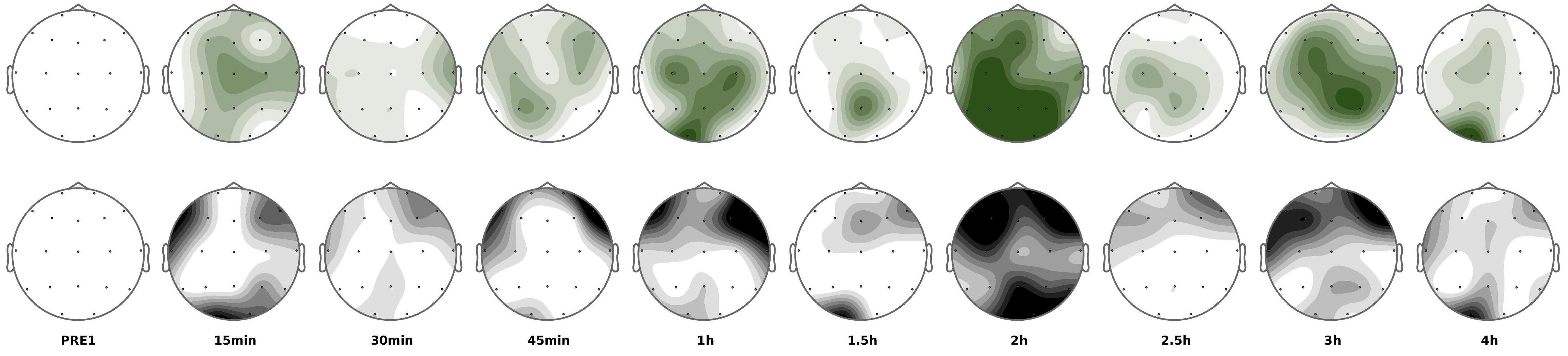


b) Directionality maps showing **decreases** in TE at all measured time points. Sources of decrease are depicted in green and sinks in gray-black. Note the predominance of anterior locations for sources between 1h and 4 hours after dosing.


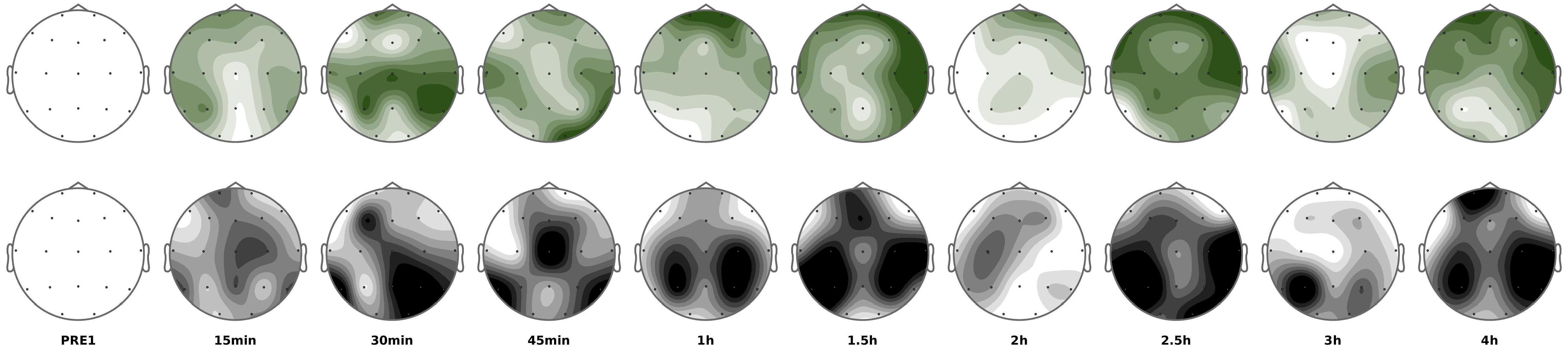


**Current source density analysis**

In addition to the TE analysis presented in the main text, we performed a current source density analysis. First, the EEG signals were subjected to a two-step artifact processing procedure (Anderer et al., 1992). It included ocular artifact minimization based on regression analysis in the time domain (Semlitsch et al., 1986), and automatic artifact rejection based on a time and frequency domain approach (Anderer et al., 1987). This was followed by spectral analysis for artifact-free 5-second epochs.

Subsequently, LORETA was used to estimate the three-dimensional intracerebral current density distribution from the voltage values recorded at the scalp (Pascual-Marqui et al., 1994). LORETA implements a three-shell spherical head model registered to the Talairach human brain atlas (Ary et al., 1981). The final solution space consisted of 2394 voxels. LORETA images represent the power (i.e., squared magnitude of computed intracerebral current density) in each of the 2394 voxels. Current density values were estimated based on the EEG cross-spectral matrix and then squared for each voxel and frequency band (Frei et al., 2001).

**Figure S2:** Effects of ayahuasca on regional cortical electrical activity at 1.5, 2 and 2.5 hours after administration. The statistical maps show voxels with significant differences in intracerebral current density (0.3-30 Hz) for the comparison between ayahuasca and placebo. Results are at p<0.05 corrected for multiple comparisons using the method described by Holmes (Holmes et al., 1996). Blue indicates significant current density decreases after ayahuasca. The characteristics of the clusters identified are provided in the table below.


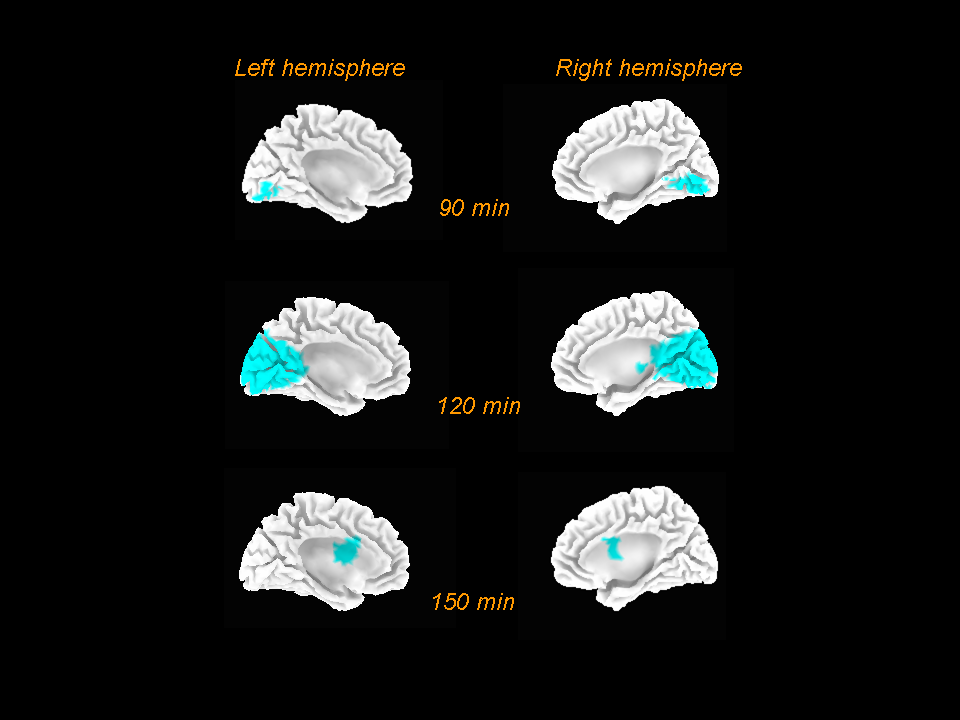


| **Time point** | **Talairach coordinates** | **Max. t value** | **Brodmann area** | **Anatomy** |
| --- | --- | --- | --- | --- |
| 90 min | 5,-83,-8 | -4.16 | 18 | Lingual gyrus |
| 120 min | 5,-77,22 | -5.61 | 18 | Cuneus |
| 150 min | -5,11,22 | -4.48 | 33/24 | Anterior cingulate |

References

Anderer P, Saletu B, Kinsperger K, Semlitsch H (1987) Topographic brain mapping of EEG in neuropsychopharmacology--Part I. Methodological aspects. Methods Find Exp Clin Pharmacol 9:371–384.

Anderer P, Semlitsch HV, Saletu B, Barbanoj MJ (1992) Artifact processing in topographic mapping of electroencephalographic activity in neuropsychopharmacology. Psychiatry Res 45:79–93.

Ary JP, Klein SA, Fender DH (1981) Location of sources of evoked scalp potentials: corrections for skull and scalp thicknesses. IEEE Trans Biomed Eng 28:447–452.

Frei E, Gamma A, Pascual-Marqui R, Lehmann D, Hell D, Vollenweider FX (2001) Localization of MDMA-induced brain activity in healthy volunteers using low resolution brain electromagnetic tomography (LORETA). Hum Brain Mapp 14:152–165.

Holmes AP, Blair RC, Watson JD, Ford I (1996) Nonparametric analysis of statistic images from functional mapping experiments. J Cereb Blood Flow Metab Off J Int Soc Cereb Blood Flow Metab 16:7–22.

Pascual-Marqui RD, Michel CM, Lehmann D (1994) Low resolution electromagnetic tomography: a new method for localizing electrical activity in the brain. Int J Psychophysiol Off J Int Organ Psychophysiol 18:49–65.

Semlitsch HV, Anderer P, Schuster P, Presslich O (1986) A solution for reliable and valid reduction of ocular artifacts, applied to the P300 ERP. Psychophysiology 23:695–703.
